# Determinants of the geographic distribution of Puumala virus and Lyme borreliosis infections in Belgium

**DOI:** 10.1186/1476-072X-6-15

**Published:** 2007-05-02

**Authors:** Catherine Linard, Pénélope Lamarque, Paul Heyman, Geneviève Ducoffre, Victor Luyasu, Katrien Tersago, Sophie O Vanwambeke, Eric F Lambin

**Affiliations:** 1 Department of Geography, Université Catholique de Louvain, Place Pasteur 3, B-1348 Louvain-la-Neuve, Belgium; 2 Research Laboratory and Reference Laboratory for Vector-borne Diseases, Queen Astrid Military Hospital, B-1120 Brussels, Belgium; 3 Scientific Institute of Public Health (IPH), Unit of Epidemiology, B-1050 Brussels, Belgium; 4 Research Group and Information on tick-borne diseases (RILY), Centre de Vaccinations, Clinique St-Pierre, B-1340 Ottignies, Belgium; 5 Research group of Evolutionary Biology, University of Antwerp, B-2020 Antwerp, Belgium

## Abstract

**Background:**

Vector-borne and zoonotic diseases generally display clear spatial patterns due to different space-dependent factors. Land cover and land use influence disease transmission by controlling both the spatial distribution of vectors or hosts, and the probability of contact with susceptible human populations. The objective of this study was to combine environmental and socio-economic factors to explain the spatial distribution of two emerging human diseases in Belgium, Puumala virus (PUUV) and Lyme borreliosis. Municipalities were taken as units of analysis.

**Results:**

Negative binomial regressions including a correction for spatial endogeneity show that the spatial distribution of PUUV and Lyme borreliosis infections are associated with a combination of factors linked to the vector and host populations, to human behaviours, and to landscape attributes. Both diseases are associated with the presence of forests, which are the preferred habitat for vector or host populations. The PUUV infection risk is higher in remote forest areas, where the level of urbanisation is low, and among low-income populations. The Lyme borreliosis transmission risk is higher in mixed landscapes with forests and spatially dispersed houses, mostly in wealthy peri-urban areas. The spatial dependence resulting from a combination of endogenous and exogenous processes could be accounted for in the model on PUUV but not for Lyme borreliosis.

**Conclusion:**

A large part of the spatial variation in disease risk can be explained by environmental and socio-economic factors. The two diseases not only are most prevalent in different regions but also affect different groups of people. Combining these two criteria may increase the efficiency of information campaigns through appropriate targeting.

## Background

Numerous human infectious diseases, particularly vector-borne and zoonotic diseases, are thought to emerge or re-emerge in some parts of the world due to environmental changes. The spatial distribution of two emerging human diseases were investigated and compared in Belgium: Puumala virus (PUUV) and Lyme borreliosis. Since the former is a zoonotic rodent-borne infection (i.e. transmissible from vertebrate animals to humans) and the latter a tick-borne infection, zoonotic and vector-borne at the same time, both strongly depend on the natural environment.

In Europe, hantaviruses (family *Bunyaviridae*) are causative agents of human zoonoses called haemorrhagic fever with renal syndrome (HFRS). Hantaviruses are transmitted via rodent excretions, unlike the other viruses from the *Bunyaviridae *family that are transmitted by arthropod vectors [1]. In western and northern Europe, Puumala virus (PUUV) causes a mild form of HFRS: nephropathia epidemica. PUUV is associated predominantly with the bank vole (*Clethrionomys glareolus*) [2-5]. Ecological factors such as favourable habitat, food supply, and climatic conditions cause interannual fluctuations in populations of bank voles.

Lyme borreliosis is an infection caused by a spirochete named *Borrelia burgdorferi*. This zoonosis is a tick-borne disease present in the northern hemisphere where the main vector species in European countries is *Ixodes ricinus *[6,7]. The complexity of disease dynamics is due to the life cycle of ticks, composed of three developmental stages (larvae, nymph, and adult). Infected ticks can transmit spirochetes to the host every time they take a blood meal, which occurs once per stage. Each of the components of the system (spirochete, tick vector, and hosts) is influenced by external biotic and abiotic factors [8]. Consequently, human activity can directly or indirectly influence the disease ecology by its impact on landscapes and host populations.

**Figure 1:**
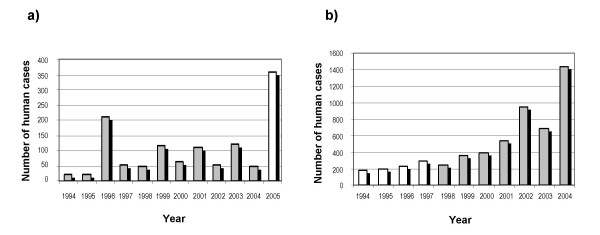
**Number of human infections over the last decade in Belgium**. (a) PUUV human cases between 1994 and 2005. (b) Lyme borreliosis infections between 1994 and 2004. Only data in dark were used in statistical analyses. Data source: Institute of Public Health (IPH).

PUUV and Lyme borreliosis infections followed different evolutions in Belgium over the last decade. PUUV human infections display interannual fluctuations that follow that of their carrier rodent population [9-11], with distinctive epidemic years in 1996, 1999, 2001, 2003 and 2005. By contrast, Lyme infections show a large linear increase. This may be due in small part to a better knowledge, diagnostic, and census of the disease but this is unlikely to fully explain the reported increase [12] (Figure 1).

**Figure 2:**
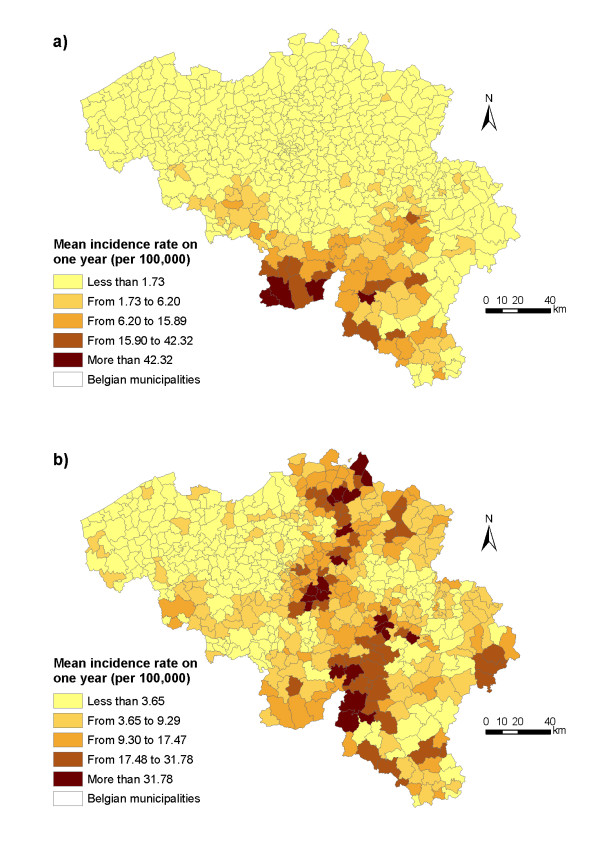
**Spatial variation in incidence rates of human infections in Belgium**. (a) Spatial distribution of PUUV mean annual incidence rates per municipality for the 1994–2004 period. (b) Spatial distribution of Lyme borreliosis mean annual incidence rates per municipality for the 1998–2004 period. Discretization method: natural breaks (Jenks). Data source: Institute of Public Health (IPH).

The spatial distributions of both diseases in Belgium are also dissimilar but both are highly clustered in space (Figure 2). Hantavirus human cases concentrate along the French-Belgian border, in the provinces of Hainaut, Namur and Luxembourg. This concentration is particularly high during non-epidemic years, while human infections slightly spread to other parts of Belgium during epidemic years [9]. The exceptionally large number of hantavirus human infections in Belgium in 2005 (Figure 1), particularly in the province of Liège, was simultaneously observed in France and Germany [13]. The spatial distribution of Lyme borreliosis infections displays high concentrations along a North-South axis, in the provinces of Antwerp, Brabant and Namur.

Vector-borne and zoonotic diseases generally display clear spatial patterns that can be due to different space-dependent factors: (i) the limited pathogen dispersal, (ii) the spatial distribution of vectors, hosts and reservoirs, and (iii) the human exposure to the infectious agent. Ostfeld et al. (2005) define spatial epidemiology as "the study of spatial variation in disease risk or incidence" [14]. Land cover – defined by the attributes of the earth's land surface and immediate subsurface, including biota, soil, topography, surface and groundwater, and human structures – and land use – defined by the purposes for which humans exploit the land cover – influence directly the spatial variation in disease risk by controlling the different agents of the disease transmission process (pathogen, vectors and hosts, including humans) and the degree of contact between these agents [15].

Geographical information systems (GIS) and remote sensing are appropriate tools to describe the spatial distribution of infectious diseases and predict disease risk [16-20]. These tools have already been used to explain or predict tick-borne diseases [21-24] and rodent-borne diseases [25,26]. These previous studies were aimed at relating spatial data on land cover and climate to the ecology of vectors or hosts.

The objective of this study was to combine environmental and socio-economic factors to explain the geographic distribution of disease incidence of Puumala virus and Lyme borreliosis in Belgium. We used multivariate statistical analysis to explain the number of PUUV and Lyme borreliosis infections per municipality according to variables related to land cover, land use, settlement characteristics, socio-economic variables, risk behaviours, and host population. We used the disease incidence as proxy for disease risk because: (i) data on human infections are better inventoried than data on hosts (in the case of PUUV) or vectors (in the case of Lyme borreliosis), and (ii) human infection results from the presence of vectors/hosts and the pathogen but also from people's exposure to vectors/hosts [27]. Human exposure is generally not taken into account in studies of vector or host ecology (but see [28]).

## Data and methods

### Dependent variable

The surveillance of Lyme borreliosis and hantavirus infections is carried out through the Scientific Institute of Public Health (IPH) sentinel laboratory network. As the diagnosis of these infections is complex and needs to be confirmed by a specialised laboratory, sentinel laboratories are asked to send positive samples to the Reference Laboratories for confirmation. These Reference Laboratories (e.g., the Reference Laboratory for Vector-borne diseases in Brussels) send monthly data on confirmed cases to the IPH and duplicated records are eliminated. The IPH collects data per postal code, which is a subdivision of the Belgian municipality. As socio-economic census data are only publicly available at the level of municipalities, we simply aggregated data from postal codes to municipalities and conducted the study at that level.

The number of human infections was used as dependent variable and the population number was added as offset variable in the model. Therefore, the model explains the incidence rate per municipality, as it was done in [29], under the assumption that incidence and disease transmission risk are highly correlated [14]. For Puumala virus, we considered all the human infections that were diagnosed in Belgium between 1994 and 2004. For Lyme borreliosis infections, we only consider the period 1998–2004 given data availability.

The data collection method was slightly different for the two diseases. While PUUV patients were georeferenced according to their residential municipality in the IPH database, Lyme borreliosis patients were georeferenced according to the municipality where they thought the contamination took place. Since, in most cases, Lyme contamination by a tick took place close to one's house, we assumed that the municipality of contamination that was recorded is the municipality of residence. We thus considered municipalities of residence for both diseases. This assumption allows combining socio-economic and environmental characteristics of municipalities in explaining disease occurrence.

### Covariates

Independent variables included several factors that have been related to the spatial variation in disease risk, including proxies for human behaviours that facilitate contacts with pathogenic agents (via vectors, hosts or a contaminated environment) (Table 1).

**Table 1:** Summary statistics of dependent and independent variables

**Dependent variables**					
Label	Variable	Mean	Std. Dev.	Min.	Max.

PUUV	total number of PUUV infections during the 1994–2004 period	1.5	5.3	0	88
Lyme	total number of Lyme borreliosis infections during the 1998–2004 period	7.8	13.5	0	104
					
**Independent variables**					

Label	Variable	Mean	Std. Dev.	Min.	Max.

*Land cover and land use*					
propforest	proportion of forest area (%)	13.7	17.7	0.0	83.5
propleaf	proportion of broad-leaved forest area (%)	5.3	8.2	0.0	54.9
urbanisation	urbanisation level from a morphological point of view (5 categories)	3.0	1.0	1.0	5.0
*Settlement characteristics*					
housesep	proportion of people living in a separated house (%)	50.3	20.3	0.7	87.2
housetwin	proportion of people living in a twinned house (%)	20.6	6.6	1.0	42.0
houseadj	proportion of people living in an adjoining house (%)	18.9	12.5	1.9	59.2
apartment	proportion of people living in an apartment (%)	8.8	11.7	0.2	71.6
cottage	proportion of people living in a caravan or country cottage (%)	0.2	0.5	0.0	8.0
*Socio-economic data*					
income	average income per 1000 inhabitants in 2002	25173	3752	16916	39735
pop94_04	average population for the 1994–2004 period	17360	27585	86.82	451778
pop98_04	average population for the 1998–2004 period	17473	27492	85.57	448782
*Risk behaviours*					
hunting	proportion of people with a hunting licence for Flemish or Walloon forests during the hunting year 2004–2005 (%)	0.34	0.29	0.00	3.45
*Host population*					
roedeer	density of roe deer (heads/km^2^)	1.4	2.0	0.0	18.2

#### Land cover and land use

Land cover determines the spatial distribution of vectors, hosts and animal reservoirs according to their habitat preferences. Broad-leaved forests, being considered as the favourite habitat for bank voles and ticks alike are expected to have an impact on disease risk. The CORINE Land Cover 2000 database, with a spatial resolution of 100 meters, was produced between 1999 and 2001 on the basis of satellite image interpretation [30]. The proportion of municipality area covered by forests (categories 3.1.1, 3.1.2 and 3.1.3 from CORINE Land Cover 2000) and broad-leaved forests (category 3.1.1 from CORINE Land Cover 2000) were extracted.

Land use determines human presence in a given place (settlement, leisure, agriculture,...) at particular times of the year and of the day. In built-up areas, the presence of vectors and hosts is very constrained ecologically and disease risk is expected to be low. A higher disease risk is expected in landscape mosaics of human-dominated and natural covers, where contacts between humans and vectors or hosts are more frequent [29]. To test the influence of the level of urbanisation, we used a classification based on the 1991 survey of the Directorate-general Statistics Belgium that classifies municipalities in 5 categories according to their morphological urbanisation level: central municipalities of main towns; high, medium and low level of morphological urbanisation; and rural municipalities [31]. The morphological urbanisation level of municipalities was determined using the population density and the proportion of built-up areas.

#### Settlement characteristics

Settlement density and types are expected to have an impact on the likelihood of contacts between humans and vectors or hosts. People living at the vicinity of forests are more likely to be infected because they are more often in direct contact with soil and wood potentially infected by PUUV. For some PUUV human cases in France, the only risk factor found was the proximity between habitation and forest [32]. Outdoor activities, even in the garden, also increase the probability of being bitten by a tick. The 2001 socio-economic survey of the Directorate-general Statistics Belgium provides data on population distribution according to residential characteristics [33]. Habitations are divided in 5 categories: separated houses, twinned houses, adjoining houses, apartments, and mobile homes or country cottages. We used the proportion of people living in these different settlement types for each municipality.

#### Socio-economic data

Certain professional or leisure activities that are dominant in certain municipalities could be reflected in the socio-professional level. For example, municipalities with many farmers and forest workers, such as loggers or foresters, which are more exposed to disease risk, generally correspond to lower mean incomes. By contrast, peri-urban zones, characterized by large gardens surrounded by woods generally correspond to higher incomes. A higher income may also enhance the propensity to consult a doctor and therefore the probability to detect the disease. Since 2000, the Directorate-general Statistics Belgium performs an annual survey on Belgian household budgets. We used data on average taxable income per tax return form in each municipality from 2002, the most recent publicly available data [34]. Data on the number of inhabitants per municipality are also available for 1993 and 2000 to 2004. Data were interpolated for missing years to estimate the average number of inhabitants for the period 1994–2004 for PUUV and 1998–2004 for Lyme borreliosis.

#### Risk behaviours

Specific human behaviours are expected to increase risk for the two diseases. Risk activities for PUUV include working in forest, farming, animal trapping, woodcutting or reopening rodent infested buildings [2,10,32], and are mainly linked to professional activities [2]. Lyme borreliosis incidence is more linked to peridomestic and recreational activities such as gardening, hiking or other outdoor activities. Few data on these behaviours are available. A detailed analysis of risk behaviours would require data at the individual level rather than municipalities, as it was done in [2,5]. In this study, we included only the proportion of people who were holding a hunting licence for Flemish or Walloon forests during the hunting year 2004–2005. These data were provided at the municipality level by institutions that deliver licences to hunt either in Walloon forests (data provided by the "Nature and forest" Division of the Walloon Region, Hunting and Fishing direction, 2005) or in Flemish forests (data provided by district police stations, Nature and Forest agency of the Flanders Region, 2006) and were associated with the municipality of residence. We assumed that people have only one licence, to hunt either in Walloon or Flemish forests.

#### Host population

The roe deer, the main host of *Ixodes ricinus *– although not a reservoir – plays an important role of maintenance and co-feeding for ticks [35,36]. Roe deer abundance should therefore be related to tick abundance and Lyme borreliosis transmission risk, as it was verified in Denmark [37]. The absence of deer could also amplify tick abundance at small spatial scales [38]. Censuses on the number of roe deer (estimation of the number of roe deer on feet) are compiled by regional institutions in Belgium. Data were provided by: (i) the "Instituut voor Bosbouw and Wildbehher" (IBW), "Wildbeheer" department, "afschot reewild" for Flanders, (ii) the "grand gibier" statistics from the "Nature and forest" Division of the Walloon Region, and (iii) the Brussels Environment (IBGE-BIM) for Brussels [39]. Data on roe deer abundance are available at the province level in Flanders and at the forest district level in Wallonia and Brussels. These data were spatially disaggregated at the level of municipalities to be compatible with the scale of the dependent variable. For Wallonia, we first overlaid forest district and municipality boundaries in a GIS. Deer populations were allocated to each polygon resulting from the intersection between forest districts and municipalities according to the proportion of forested area of the forest district belonging to the municipality. The number of deer per municipality was then obtained by aggregation of the different polygons in a municipality. For Flanders, as municipalities are fully included in provinces, we simply allocated a deer population to municipalities in proportion to the forest area of each municipality. For Brussels, we only had an estimate of a density of 3 roe deer for 100 hectares of forest [39]. Using this estimate and the proportion of forest area in each municipality of Brussels, we computed the expected number of deer per municipality. For the three regions, we considered the average number of deer over the 1998–2004 period. This variable was used only in the model for Lyme borreliosis.

### Statistical distributions analysis

**Figure 3:**
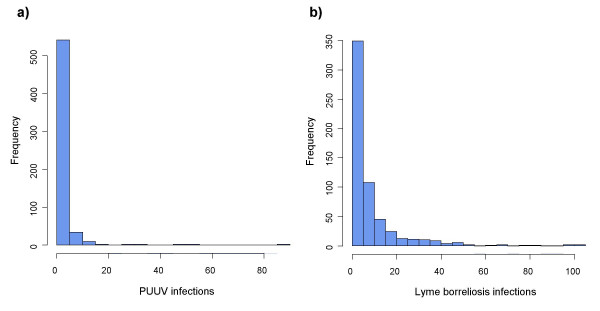
**Frequency charts of dependent variables**. (a) Number of PUUV infections per municipality for the 1994–2004 period. (b) Number of Lyme borreliosis infections per municipality for the 1998–2004 period.

Count data such as the number of human infections often follow a Poisson distribution. This distribution requires that the mean is equal to the variance, a condition commonly not fulfilled. Our data follow such an extra-Poisson model. The dependent variables (total number of human infections) for both diseases have highly asymmetrical statistical distributions (Figure 3). Variances are much larger than the means of the number of human infections for both distributions. For PUUV infections, more than half of the municipalities had no reported cases during the ten years period. Lyme borreliosis infections are more evenly distributed, with only 95 out of 589 municipalities (16%) with no case. In terms of incidence rates, the maximum annual PUUV incidence rate reaches 90.2 for 100,000 inhabitants in the municipality of Chimay. For Lyme borreliosis, four municipalities have a mean incidence rate exceeding 50 (Kasterlee, Court-St-Etienne, Dinant, and Beauraing).

As we are interested in inference of regression parameters, the use of a model that allows for the possibility of extra-Poisson variation is preferable [40]. The negative binomial probability distribution is a generalisation of the Poisson distribution with variances higher than means. An overdispersion parameter k (≥ 0) is added:

var(*Y*) = *μ *+ k * *μ*^2^

When k = 0, the negative binomial distribution is reduced to a Poisson distribution.

Likelihood ratio tests and Dean's tests for overdispersion were performed with the DCluster package of the R software. The three tests were very significant and the null hypothesis of overdispersion was not rejected (Table 2).

**Table 2:** Likelihood ratio test and Dean's tests for overdispersion

	**PUUV**	**Lyme borreliosis**
Likelihood Ratio Test	398.33*	2367.71*
Dean's P_B test	45.99*	134.66*
Dean's P'_B test	46.35*	134.91*

* P-value < 0,0001

### Multivariate analyses

#### Non-spatial model

Generalized linear regression models were computed using the R statistical software. All variables were relative to the population size N_i _of municipalities and the logarithm of N_i _was added as an offset variable in the models. The distribution parameter k is estimated by the maximum likelihood method, as the other parameters of the negative binomial regression model. The Akaike's information criterion (AIC), which takes the number of estimated parameters into account, was used to select the covariates to retain in the model. Variables were tested for collinearity and confounding factors.

#### Spatial model

Spatial autocorrelation appears when a spatial dependence due to neighbouring effects (i.e., spatial externalities) exists between spatial entities. Spatial dependence usually results from a combination of exogenous and endogenous processes. In an exogenous spatial process, the identified spatial pattern is generated by independent factors, whereas endogenous spatial dependence is an inherent property of the variable of interest [41]. Spatial epidemiological models are particularly prone to spatial autocorrelation since disease transmission requires contacts between infected and susceptible agents. The infection rate in a locality is therefore influenced by the infection rates in neighbouring spatial entities. The spatial diffusion of epidemic diseases and the influence on infection of many unobserved factors also cause spatial autocorrelation.

**Figure 4:**
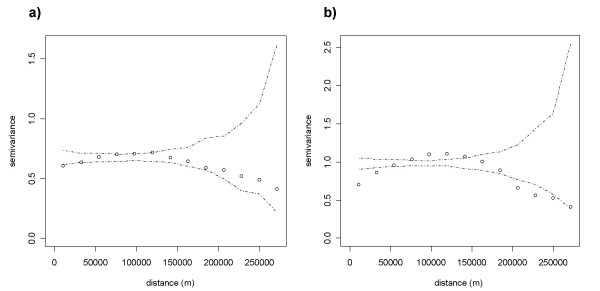
**Empirical variograms and envelopes of residuals from non-spatial models**. (a) Residuals from non-spatial negative binomial regression on PUUV infections. (b) Residuals from non-spatial negative binomial regression on Lyme borreliosis infections. Envelopes were computed by permutation of the data values on the spatial locations [43].

Since spatial dependence between observations skews parameter estimation, the presence of residual spatial dependence in the error term needs to be examined [42]. Empirical variograms and their envelopes were estimated with the geoR package from the R software (Figure 4) [43]. The envelopes of empirical variograms were computed based on a simple Monte Carlo test that permutes data values on spatial locations. Therefore, evidence of spatial dependence is noticed when points representing residual values fall outside the envelope on an empirical variogram [44].

For linear regression models, one can distinguish between endogenous and exogenous spatial processes by comparing spatial lag and spatial error models [45]. This approach does not apply for generalized linear models. For negative binomial regressions, endogenous spatial dependence can be corrected for by adding an instrumental variable that explains the dependent variable in the neighbouring municipalities [46]:

log(*Y*_*i*_) = *α *+ *β*_1_*x*_*i*1 _+ *β*_2_*x*_*i*2 _+ ... + *β*_*n*_*x*_*in *_+ *λ*Y^Ej
 MathType@MTEF@5@5@+=feaafiart1ev1aaatCvAUfKttLearuWrP9MDH5MBPbIqV92AaeXatLxBI9gBaebbnrfifHhDYfgasaacH8akY=wiFfYdH8Gipec8Eeeu0xXdbba9frFj0=OqFfea0dXdd9vqai=hGuQ8kuc9pgc9s8qqaq=dirpe0xb9q8qiLsFr0=vr0=vr0dc8meaabaqaciaacaGaaeqabaqabeGadaaakeaacuWGzbqwgaqcamaaBaaaleaacqWGfbqrcqWGQbGAaeqaaaaa@3093@ + *σε*_*i*_

where Y_i _is the expected value of the dependent variable y for the municipality i, *x*_in _are the dependent variables with their associated regression coefficients *β*_*n*_, *σε*_*i *_is the disturbance term and Y^Ej
 MathType@MTEF@5@5@+=feaafiart1ev1aaatCvAUfKttLearuWrP9MDH5MBPbIqV92AaeXatLxBI9gBaebbnrfifHhDYfgasaacH8akY=wiFfYdH8Gipec8Eeeu0xXdbba9frFj0=OqFfea0dXdd9vqai=hGuQ8kuc9pgc9s8qqaq=dirpe0xb9q8qiLsFr0=vr0=vr0dc8meaabaqaciaacaGaaeqabaqabeGadaaakeaacuWGzbqwgaqcamaaBaaaleaacqWGfbqrcqWGQbGAaeqaaaaa@3093@ is the linear prediction of the dependent variable in neighbouring spatial entities j. This model was implemented in two stages, following the method of Mallar (1977) [46]. Firstly, a separate negative binomial regression model, with the number of human infections in the surrounding municipalities as dependent variable, provided linear predictions of *Y*_*Ej *_in each municipality. Covariates included the same variables as for the non-spatial model computed for both the central and its neighbouring municipalities. Interaction variables were also included to maximize the performance of the model. In the second stage, these linear predictions Y^Ej
 MathType@MTEF@5@5@+=feaafiart1ev1aaatCvAUfKttLearuWrP9MDH5MBPbIqV92AaeXatLxBI9gBaebbnrfifHhDYfgasaacH8akY=wiFfYdH8Gipec8Eeeu0xXdbba9frFj0=OqFfea0dXdd9vqai=hGuQ8kuc9pgc9s8qqaq=dirpe0xb9q8qiLsFr0=vr0=vr0dc8meaabaqaciaacaGaaeqabaqabeGadaaakeaacuWGzbqwgaqcamaaBaaaleaacqWGfbqrcqWGQbGAaeqaaaaa@3093@ were included among the explanatory variables in the negative binomial regression.

## Results

### Non-spatial model

Both non-spatial regression models have a high explanatory power, with all parameters being very significant and a residual deviance value close to the number of degrees of freedom (DF) (with a ratio of 0.81 for PUUV and 1.1 for Lyme), indicating a good model fit. For PUUV, we reduced the deviance from 1,167.8 for the null model to 474.9 (with 584 DF). For Lyme, it was reduced from 857.9 to 642.4 (with 584 DF) (Tables 3 and 4). Each model includes four significant explanatory variables. The dispersion parameters for the negative binomial regressions (0.77 for PUUV and 1.20 for Lyme borreliosis) were taken to be 1.

**Table 3:** Parameter estimates of significant variables using negative binomial regressions on PUUV infections (1994–2004)

	**Non-spatial model**	**Spatial model**
**Parameter**	**Estimate**	**Estimate**

Intercept	-7.982***	-9.308***
propleaf	0.0915***	0.0469***
income	-0.0001***	-0.00006**
urbanisation	-0.192***	-0.246**
hunting	189.6*	152.0***
Linear predictors in neigbourhood municipalities		0.5491***
		
Degrees of freedom	584	583
Null deviance	1167.8	1311.9
Residual deviance	474.9	484.3
AIC	1418.3	1389.4
2 × log-likelihood	-1406.3	-1375.4

*** P-value < 0,0001; ** P-value < 0,01; * P-value < 0,1

**Table 4:** Parameter estimates of significant variables using negative binomial regressions on Lyme borreliosis infections (1998–2004)

	**Non-spatial model**	**Spatial model**
**Parameter**	**Estimate**	**Estimate**

Intercept	-10.41***	-11.08***
propforest	0.0225***	0.0254***
income	0.0001***	0.00004***
housesep	0.0084***	0.0098***
roedeer	0.0955***	0.0302
Linear predictors in neigbourhood municipalities		0.3675***
		
Degrees of freedom	584	583
Null deviance	857.9	916.6
Residual deviance	642.4	642.3
AIC	3220.9	3182.9
2 × log-likelihood	-3208.9	-3168.9

*** P-value < 0,0001

The variograms of residuals for these non-spatial regressions lead to a different diagnostic for the two diseases (Figure 4). For PUUV, the envelope contains all the points of the empirical variogram. This suggests that the spatial dependence in PUUV incidence is mainly due to an exogenous process that is associated with one or several explanatory variables included in the model, which have their own spatial structure. The proportion of broad-leaved forests, one of the significant independent variables, explains a large part of this spatial dependency in PUUV incidence. By contrast, for Lyme borreliosis, the residuals of the regression model display a significant spatial dependence. This could be caused by unobserved explanatory variables, i.e. an external process whose spatial structure is not represented in the model. It could also be due to an endogenous effect if the incidence of Lyme borreliosis in one location influences its incidence in neighbouring locations.

### Spatial model

To test for spatial endogeneity in the Lyme borreliosis model, a linear predictor of the dependent variable in neighbouring municipalities was estimated. For comparative purpose, the same approach was applied to the PUUV model, even though the spatial dependency was already reduced in the non-spatial model for this disease. Both spatial models have a high explanatory power and very significant parameters (Tables 3 and 4). For PUUV, the residual deviance value is closer to DF compared to the non-spatial model, with a ratio that reaches 0.83. For Lyme, the gap between the residual deviance and DF is similar to the non-spatial model (with a ratio of 1.1). Each model includes the same explanatory variables as the non-spatial models. All these covariates – except for roe deer density for Lyme – remain significant and with the same signs. The linear predictors of the dependent variable in neighbouring municipalities are very significant in both models.

**Figure 5:**
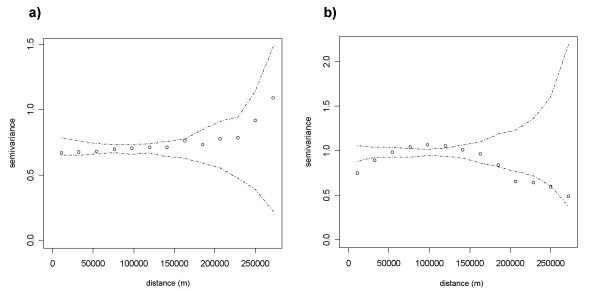
**Empirical variograms and envelopes of residuals from spatial models**. (a) Residuals from spatial negative binomial regression on PUUV infections. (b) Residuals from spatial negative binomial regression on Lyme borreliosis infections. Envelopes were computed by permutation of the data values on the spatial locations [43].

**Figure 6:**
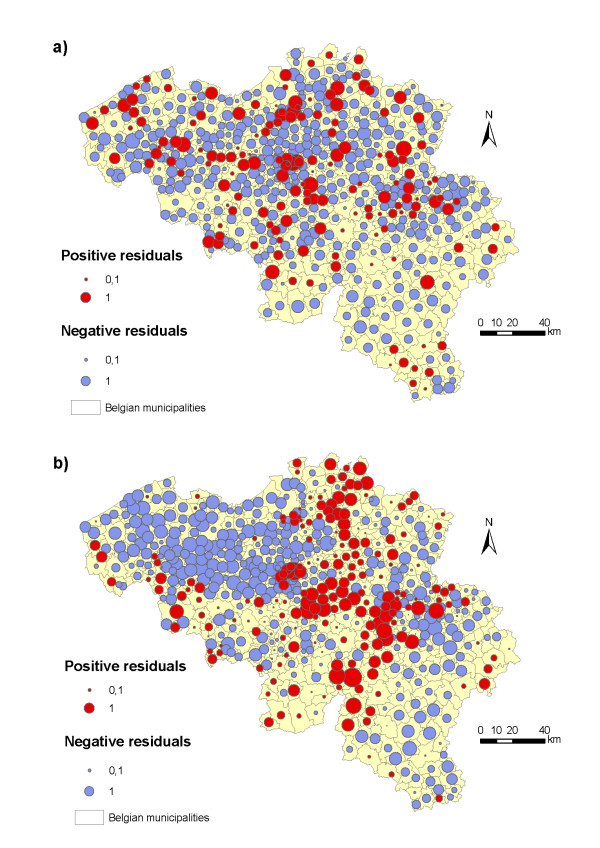
**Spatial distribution of residuals from spatial models**. (a) Residuals from spatial negative binomial regression on PUUV infections. (b) Residuals from spatial negative binomial regression on Lyme borreliosis infections.

The variograms of the residuals from the spatial regressions show that the spatial dependence is further reduced for PUUV (Figure 5). Spatial autocorrelation in PUUV incidence therefore results from exogenous – mainly via broad-leaved forest habitats – but also endogenous processes. As the envelope completely contains the points of the empirical variogram, we do not reject the hypothesis of spatial independence in the error terms of the spatial regression. By contrast, for Lyme borreliosis, even though the spatial dependence is slightly reduced in the spatial regression (Figure 5), it is still significant. Removing the roe deer density variable from the spatial model on Lyme disease did not modify the explanatory power of the model (AIC of 3181.9) nor did it reduce spatial autocorrelation. Spatial dependence in Lyme borreliosis incidence is therefore mainly due to unobserved exogenous processes that are not accounted for in the model [42]. The spatial distribution of residuals provides insights on the spatial structure of missing external factors (Figure 6). The model on Lyme disease tends to overestimate values in low-incidence areas and underestimate values in high-incidence areas (Figures 2b and 6b).

## Discussion

The spatial model on PUUV is composed of four variables, each with the expected sign, in addition to the spatial dependency term that is also significant. Two variables are related to the landscape (proportion of the area of the municipality occupied by broad-leaved forests and urbanisation), one to a socio-economic characteristic of the population (income), and another one to a risk behaviour of humans (hunting). The PUUV incidence rate is higher where the proportion of broad-leaved forests is high and where urbanisation is low. Broad-leaved forests are the favourite habitat for bank voles and are thus positively related to the disease. Moreover, bank voles use small areas and only rarely leave forests. Therefore, humans generally catch the disease in the ecological habitat of bank voles, in broad-leaved forests. A high level of urbanisation thus limits PUUV transmission. Income is negatively correlated with the disease incidence because PUUV particularly affects forest workers and farmers who are in frequent contact with bank voles or infected soil in forests or at their edge. These socio-professional categories are generally characterised by a low income. Hunters also spend time in forests and are thus particularly vulnerable to PUUV infection.

The spatial model for Lyme borreliosis infections has four explanatory variables, each of them with a positive sign, in addition to the spatial dependency term that is also highly significant. One variable is related to the host population (roe deer), two variables represent landscape attributes that favour Lyme borreliosis transmission (proportion of the area of the municipality occupied by forest and proportion of people living in separated houses), and one characterises the human population by its socio-professional level. The model confirms the hypothesis that roe deer density has an impact on spatial variations in Lyme disease transmission risk in Belgium, probably by favouring tick reproduction and co-feeding. Lyme borreliosis incidence rate is positively linked to forest cover but also to the proportion of people living in separated houses. This suggests that heterogeneous landscapes with a fragmented land use mixing forests and houses are more at risk. In areas with a high proportion of separated houses, people are more likely to have gardens and thus spend time outdoors. The presence of forests nearby further favours human-vector contacts. Peri-urbanisation could thus be one of the major causes of the recent increase in Lyme borreliosis infections. In Belgium, it largely affects Walloon Brabant and the province of Antwerp, two wealthy peri-urban areas. The positive relationship between Lyme borreliosis incidence rate and mean income supports this hypothesis since higher incomes are likely to be associated with large gardens in wooded areas and thus a larger interface between settlements and forests in peri-urban areas. Higher incomes – especially when they are associated with education level – could also increase the probability of being aware of the disease and of consulting a doctor, thus increasing the likelihood of disease detection.

The spatial distribution of PUUV and Lyme borreliosis infections are thus associated with similar factors, in particular the presence of forests. For both diseases, it is linked to the vector or host habitat preferences. Other variables influence both diseases but in opposite directions, such as mean income and urbanisation. This reflects the fact that PUUV incidence is more associated with professional outdoor activities (in low-income, rural and forested areas), whereas Lyme borreliosis is rather associated with recreational and peridomestic outdoor activities (in high income, peri-urban areas with isolated houses and forests).

Most importantly, both diseases are associated with a combination of factors linked to the vector and host populations, to human behaviours, and to landscape attributes. The latter includes both land cover (forests), as a function of vector/host ecology, and land use that determines human presence in critical habitats, the frequency of contacts between vectors and human populations, and thus human exposure to infection.

Residuals of the spatial regression model of Lyme disease still display strong spatial dependence, which suggests that one or more external factors with spatial structure are missing. However, several factors could influence and skew the spatial effect in the Lyme model. Firstly, the roe deer density data were only available at the province level in Flanders and at the forest district level in the Walloon region and Brussels. This variable was thus spatially disaggregated at the level of municipalities. Removing this variable did not reduce the spatial autocorrelation. However, representing more ecologically-relevant boundaries that separate independent deer populations (e.g., major roads) could reduce spatial autocorrelation compared to administrative boundaries [47]. Secondly, data on Lyme borreliosis incidence are based on the contamination place, whereas data on PUUV infections are based on the residential municipality. The assumption that the municipality of contamination corresponds to that of residence for Lyme disease could create a bias, especially for fine-scale analyses. This bias could increase spatial dependence effects or simply add randomly distributed errors [14]. Thirdly, information campaigns organised for doctors in certain regions could have led to a better diagnostic of Lyme borreliosis infections. High positive residuals are found for example around the municipality of Ottignies-Louvain-la-Neuve in Walloon Brabant, where an important information centre on Lyme disease – Research Group and Information on tick-borne diseases (RILY) – is located. This is highly speculative however since this municipality is also a high-income, peri-urban area with several small forests.

## Conclusion

The objective of this study was to explain the spatial distribution of a zoonotic (Puumala virus) and a vector-borne (Lyme borreliosis) disease in Belgium. The combination of environmental and socio-economic factors allowed explaining the major part of the spatial variation in disease risk. Besides the influence of land cover on the spatial distribution of vectors and hosts, land use also determines the prevalence of vector-borne and zoonotic diseases by controlling the degree of exposure of people to contact with vectors or hosts. PUUV infection risk is more prevalent in remote, low-income areas with large forests whereas the probability of Lyme borreliosis infection is higher in areas with a large interface between settlements and forests in wealthy peri-urban areas. This study showed the importance of adding variables related to human behaviour and land use in addition to land cover and habitat variables in epidemiological models. Spatial dependence diagnostics in error terms revealed a combination of endogenous and exogenous processes causing spatial clustering of incidence for both diseases. The spatial dependence could be accounted for in the model of PUUV incidence but not for Lyme borreliosis.

The results of this study have important implications to define priority areas for public health policies aimed at preventing diseases by informing the public and promoting the use of protective measures. For example, the Institute for Public Health produces information sheets on the diseases for distribution to the public. Each disease is not only most prevalent in different regions but also affects different groups of people. Combining these two criteria may increase the efficiency of information campaigns through appropriate targeting.

## Competing interests

The author(s) declare that they have no competing interests.

## Authors' contributions

CL collected data, performed the statistical analyses, interpreted the results and wrote the first draft of the manuscript. PL participated in data acquisition, analysis and interpretation. PH, GD and VL collected data on human infections. KT helped in data acquisition. SOV helped in statistical analysis and interpretation of results. EFL had the initial idea and led the design of the study, advised CL and PL, and helped to write the manuscript. All authors read and approved the final manuscript.
